# Supplementary figures and images for: Iron-mediated post-transcriptional regulation in Toxoplasma gondii
Source: PLoS Pathog. 2025 Feb 3;21(2):e1012857. doi: 10.1371/journal.ppat.1012857 (PMC11801735; doi:10.1371/journal.ppat.1012857)

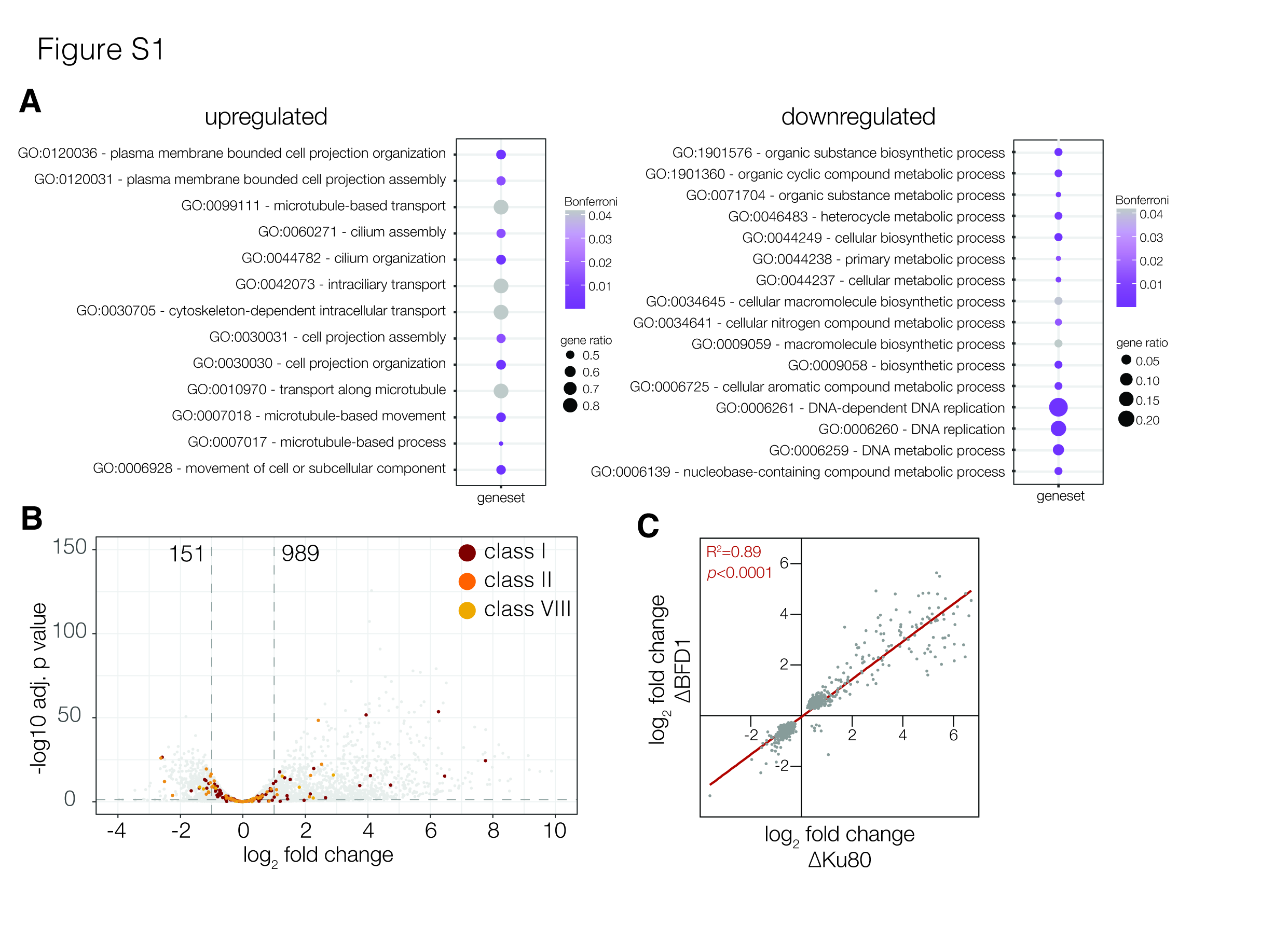

Supplement: S1 Fig — Circle size represents the proportion of genes from that GO set which were enriched in the differentially expressed genes from our data set (gene ratio). The intensity of the circles represents the significance adjusted p-value (Bonferroni corrected) of the gene ratios. This analysis was performed with ToxoDB [83]. B. Volcano plot from RNAseq data comparing RHΔBFD1 cultured in 100 µM DFO for 24 hours to standard conditions. Adjusted p-values from the Wald test with Benjamini and Hochberg correction. Cut-offs shown with dashed lines are p-adj < 0.05 and log2 fold change of >2 or <−2. C. Correlation plot showing that response of parental and ΔBFD1 to DFO treatment is highly correlated (Spearman’s correlation, R2 = 0.89, p < 0.0001). (TIF) [file ppat.1012857.s001.tif]

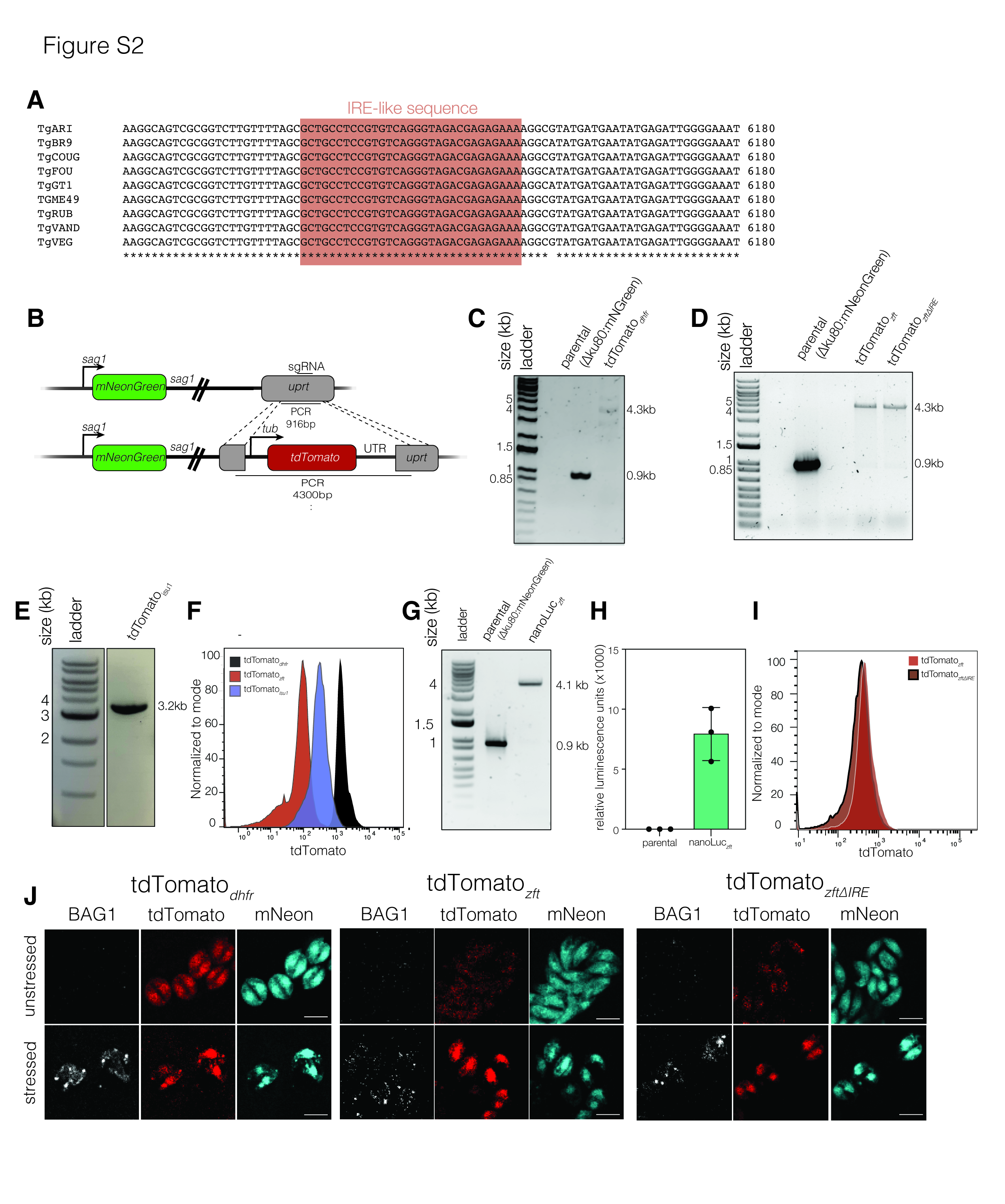

Supplement: S2 Fig — A. Alignment showing conservation of IRE sequence in the 3’UTR of zft across T. gondii strains. Alignments were performed using T-Coffee online alignment tool M-Coffee [90]. B. Schematic of the cloning strategy for adding the reporter cassettes into the uprt locus using CRISPR-Cas9 C-E. PCRs showing successful amplification of the full reporter tdTomatodhfr (C, expected size 4.3kb), tdTomatozft (D, expected size 4.3kb), tdTomatozftΔIRE (D, expected size 4.3kb), and tdTomatoisu1 (E, expected size 3.2kb), cassettes into the uprt locus of RHΔKu80:mNeonGreen parasites. Parental line included as negative control F. Overlapping histogram showing tdTomato fluorescence in untreated tdTomatodhfr (black), tdTomatoisu1 (blue) and tdTomatozft (red) reporter lines as measured by flow cytometry. G. PCR showing successful amplification of the full reporter nanoLuczft (expected size 4.1 kb). H. Luminescence experiment showing nanoLuczft expresses detectable luciferase under basal conditions. I. Overlapping histogram showing tdTomato fluorescence in untreated tdTomatozft (red) and tdTomatozftΔIRE (dark red) reporter lines as measured by flow cytometry. J. IFA images showing expression of bradyzoite marker BAG1 at 24 h post alkaline stress. Scale bar 5 µm. (TIF) [file ppat.1012857.s002.tif]

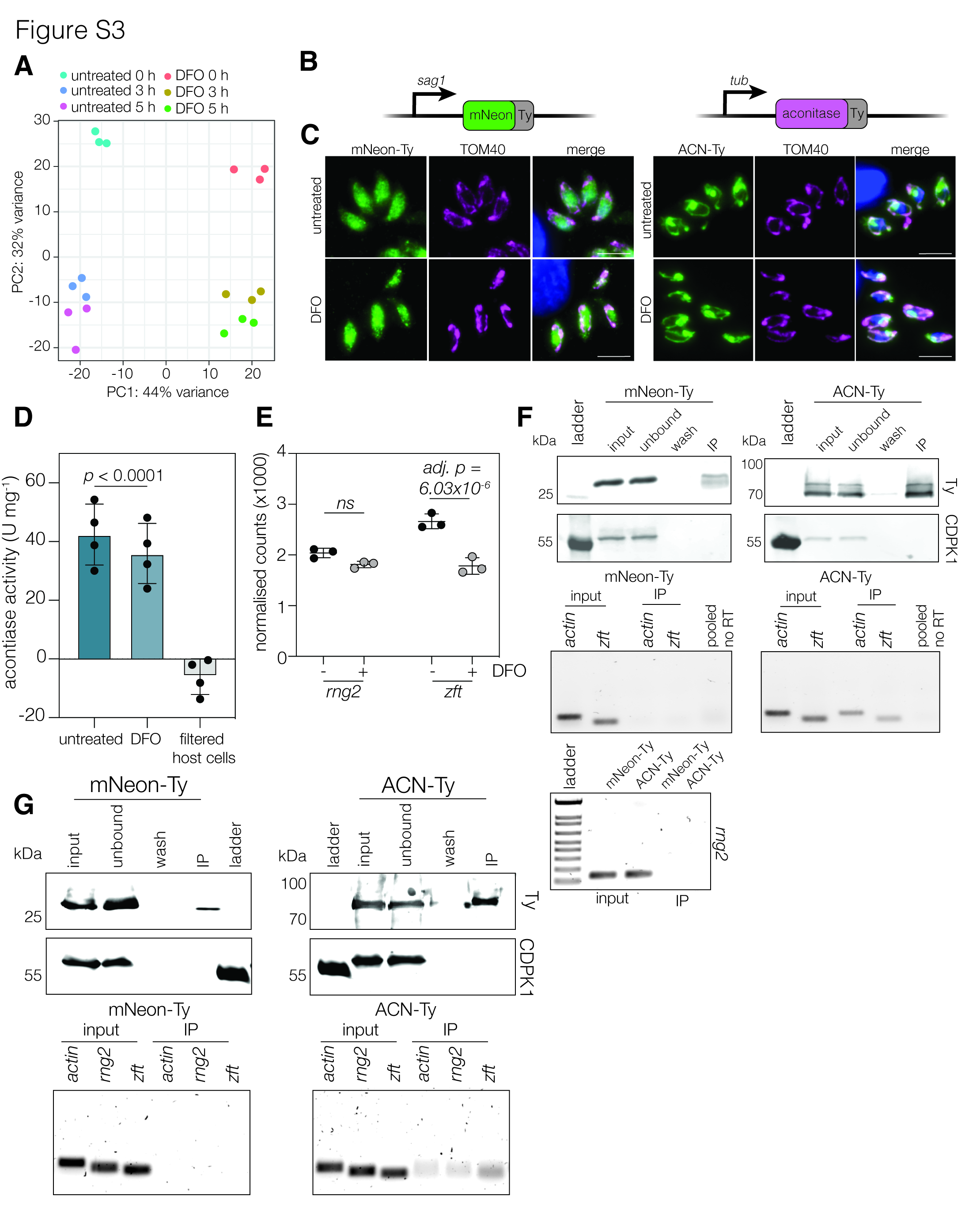

Supplement: S3 Fig — A. PCA plot showing clustering of biological replicates from mRNA stability assay. B. Schematic of tagging scheme in ΔKu80:mNeonGreen-Ty and ACN-Ty parasites. C. Immunofluorescence of ΔKu80:mNeonGreen-Ty and RHΔKu80, ACN-Ty parasites grown in standard culture or 100 µM DFO for 24 hours. Anti-TOM40 included as a mitochondrial marker and DAPI as DNA marker. Scale bars 5 µm. D. Aconitase activity assay of untreated or DFO treated parasites. Results from 4 independent experiments, ± SD. Lysed and filtered host cells are included to demonstrate minimal carryover of host aconitase activity. p value from paired t test. E. Normalised counts for zft and rng2 transcripts from RNAseq dataset comparing parasites after 24 hours treatment with 100 µM DFO, compared to untreated parasites. Points represent 3 independent experiments, bars at mean ± SD. Two (F and G) further biological replicates showing ACN-Ty interacts with zft mRNA. Blots showing the immunoprecipitation of mNeonGreen-Ty and aconitase-Ty from T. gondii parasites. Wash – the output of the 6th and final washing step. CDPK1 included as cytosolic control. From the corresponding pull down, DNA-agarose gel showing qPCR products amplified from reverse transcribed RNA from either lysates or RNA co-precipitated with mNeonGreen-Ty and ACN-Ty proteins. (TIF) [file ppat.1012857.s003.tif]

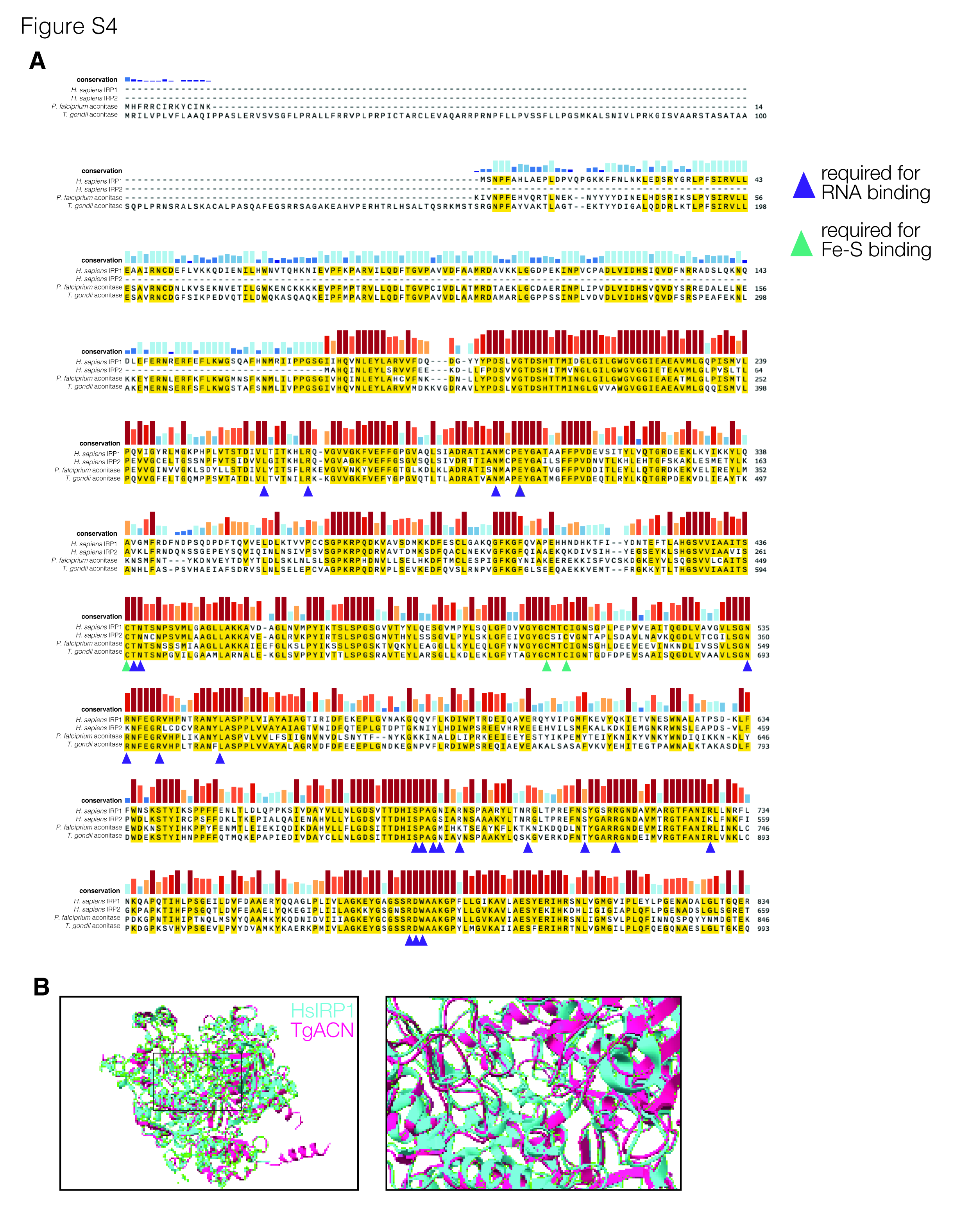

Supplement: S4 Fig — A. Amino acid sequence alignment of the Toxoplasma gondii aconitase hydratase ACN/IRP (TGME49_226730), Plasmodium falciparum aconitase hydratase (PF3D7_1342100) and human IRP1 (UniProt: P21399) and IRP2 (UniProt: P48200) in ClustalW format made using T-Coffee [90]. Green triangles indicate the residues interacting with the FeS cluster. Purple triangles indicate residues shown to interact with ferritin mRNAs in human IRP1 [61]. B. Alphafold [91,92] structure prediction showing high structural conservation between TgACN (magenta) and HsIRP1 (pdb 2B3X) (cyan), including around the FeS coordination core (yellow, inset). (TIF) [file ppat.1012857.s004.tif]

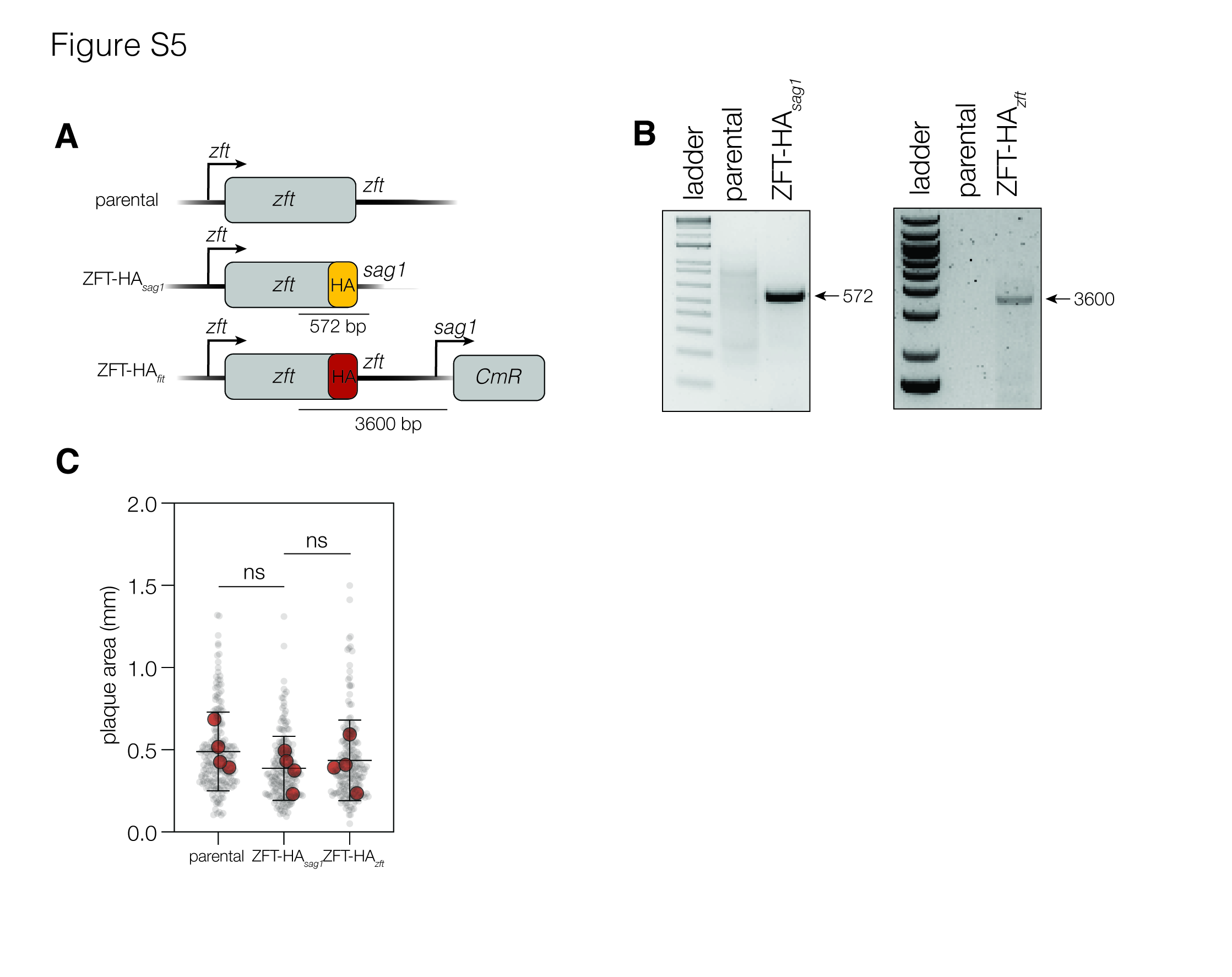

Supplement: S5 Fig — A. Schematic of tagging scheme of ZFT-HA parasites. B. PCR confirmation of tagged lines. Expected size as indicated in (A). C. Area of plaque of four independent experiments. Each point is a plaque, line at mean, ± SD. Red dots indicate average plaque size for each experiment. p > 0.05 from one way ANOVA. (TIF) [file ppat.1012857.s005.tif]
