# Supplementary material for: Iron-mediated post-transcriptional regulation in Toxoplasma gondii
Source: PLoS Pathog. 2025 Feb 3;21(2):e1012857. doi: 10.1371/journal.ppat.1012857 (PMC11801735; doi:10.1371/journal.ppat.1012857)
Supplement: Attachment — Submitted filename: Reply to reviewers.docx [file ppat.1012857.s019.docx]

We thank the reviewers for their time and attention. In reference to both their and the editors comments we have added several new experiments and significant new data, including several new RNAseq-based approaches which we have detailed below. Thanks to the comments of the reviewers, we now believe that the manuscript is now of a significantly higher standard.

**Editors comments:**

- Validate the reporter findings by mutating the endogenous 3’UTR of FIT and showing that its protein levels are no longer affected during iron depletion. –

We have assessed this question in **Figure 7A** and find that the endogenous UTR is required to stabilise protein levels at 18 h, although protein is depleted by 24h. This result was not expected and so beyond looking at endogenous protein levels, we have also shown that the change of the 3’ UTR influences parasite survival only under low iron conditions, both by growth assay and plaque assay. This provides the first evidence that regulation of the gene is important for the parasite under specific growth conditions.

Test the IRE positioning effect on its function.

We were concerned about this approach, as our results suggest that the mRNA stability and genomic context also influence our results. Instead, we have significantly expanded our work on mRNA stability, by qPCR and RNA-seq approaches and added these into **Figure 6 and table S12**. We have expanded on this below.

Demonstrate that ACN is binding to the IRE transcripts by performing either more stringent IPs or a high throughput sequencing of the transcripts pull-down in the IP.

We have done this, and found no evidence of a direct interact by sequencing the IP transcripts, new data in **figure 6** and **table S11**. While this does not exclude ACN from an RNA-binding role in *Toxoplasma*, we now do not believe that this is mediating the change in mRNA stability we see in our parasite reporter line.

Address the concerns about statistical analyses

We addressed this, please see below for details

Reviewer's Responses to Questions

**Part I - Summary**
Please use this section to discuss strengths/weaknesses of study, novelty/significance, general execution and scholarship.

Reviewer #1: This manuscript addresses mechanisms by which the parasite Toxoplasma gondii responds to depleted iron levels. Results show that Toxoplasma experiences changes in gene expression when iron is scare and uses the post-transcriptional IRE/IRP system, which is well-characterized in a number of other eukaryotes and has been described for Plasmodium. The authors complete an impressive suite of well-controlled experiments to show conservation of this regulatory pathway in Toxoplasma.

Reviewer #2: This is a manuscript from Sloan et al. entitled "Keeping FIT: Iron-mediated post-transcriptional regulation in Toxoplasma gondii". It describes the identification and characterization of an Iron Response Element (IRE) in the 3'UTR of the TGME49_261720 transcript using a reporter system. Regulation of gene expression by Iron has been shown recently by the same group. However, the molecular mechanisms underlying this regulation have not been explored so far. Although IREs are widespread in eukaryotes and their sequence is relatively conserved, their functionality in the parasite was not investigated. This work therefore explores a regulation mechanism that seems common to eukaryotes. The authors provide convincing data on the functionality of the IRE found in the 3'UTR of the TGME49_261720 transcript in a heterologous reporter system. However, only one of the 142 IREs that were found in transcripts is functional (out of 2 tested). Moreover, the authors fell short of confirming the role of Aconitase in this regulation, a key conserved mechanism in Iron response.

Reviewer #3: this is an interesting paper about a topic that has not been well explored. data are well presented and organized. I only have minor comments. Nice work.

**Part II – Major Issues: Key Experiments Required for Acceptance**
Please use this section to detail the key new experiments or modifications of existing experiments that should be absolutely required to validate study conclusions.

Generally, there should be no more than 3 such required experiments or major modifications for a "Major Revision" recommendation. If more than 3 experiments are necessary to validate the study conclusions, then you are encouraged to recommend "Reject".

Reviewer #1: Major concern:
1. There is no evidence that FIT protein levels increase in an IRE-dependent way. Endogenous FIT should be epitope-tagged using its native 3’UTR or its 3’UTR deleted of its IRE sequence. This experiment seems necessary to verify the conclusions drawn from the reporter assays and validate the model proposed by the authors.

We have addressed this concern by switching the endogenous UTR of zft with sag1 and assessing protein levels and parasite survival. We show that the presence of the endogenous UTR is required for maintenance of the protein at 18 h, although protein levels are decreased by 24 h. This mirrors qPCR-results, although not necessarily the tdTomato reporter which showed higher protein levels even at 24h. We suggest that this is due to a faster protein turn over level of ZFT which we will be investigating in future studies. Further, we have modified the language around the IRE throughout and now suggest that the IRE is important for the function of the zft UTR, but that other features of the UTR may also play an important role.

Suggested experiments to improve study and strengthen conclusions:
1. Transcriptomics data suggest that iron deprivation induces bradyzoite differentiation (Fig. 1). Authors should consider a simple differentiation assay to confirm signature features of tissue cyst formation (and at what frequency) during iron deficiency. This is important not only to bolster the author’s claim but also to assess whether iron depletion may be a better inducer of in vitro bradyzoite development than current methods.

This experiment was recently published by two independent groups (Renaud et al, 2024, Ying et al 2024) where they demonstrate conclusively that iron deprivation leads to differentiation. For this reason, we have chosen not to repeat the experiments here, but instead reference the relevant work. However, to examine the transcriptional aspects in more detail, here we make use of a parasite line with a deletion in the master regulator of bradyzoite differentiation, BFD1. We show that under iron deprivation, we still see upregulation of bradyzoite-related genes although to a lesser (although still significant) extent, suggesting that iron deprivation is triggering the start of the bradyzoite programme, even in the absence of the master regulator. The mechanism behind this surprising result is beyond the scope of the current paper.

2. Chelation of other metals serves as a nice control to iron deprivation (Fig 3); it may be of interest to also test if a different inducer of bradyzoite development activates the FIT reporter as a further control on its specificity.

Thank you for this suggestion, we have added this data at **Figure 5E and F.** We do see induction of the reporter, but to a lower level than iron depletion. This change could be due to increased cellular stress activating the reporter, or alternatively it is possible that the differentiation into bradyzoites results in a decrease in available iron to the parasite. We hope to investigate this further in future studies.

3. The idea that IRE position effects its function is an interesting hypothesis; authors may want to consider designing a reporter to formally test this (i.e. move the IRE-like sequence of iscu closer to the stop codon). Alternatively (or in addition), authors could consider a reporter that moves the IRE sequence of FIT further downstream in the ’UTR.

This is an intriguing idea, however at this point we have not experimentally assessed the functional role of IRE positioning. Please see below our response to reviewer 2 for more details

4. An independent line of evidence would strengthen the conclusion that ACN binds to zft at the IRE. For example, immobilizing the TGME49_261720 3’UTR with and without the IRE and passing lysate from ACN-Ty expressing parasites over it.

We agree and have addressed this in our response the editor above.

Reviewer #2: Major concerns

The fit transcript is downregulated in the absence of iron. How do the authors reconcile their data on the functionality of this IRE with this measured expression in the absence of Iron?

We have addressed this apparent discrepancy in our results in the discussion. The downregulation of transcript abundance in our initial RNAseq dataset is likely a reflection of the comparatively late timepoint at which the experiment was performed (after 24 hours of iron deprivation). The iron-responsive properties of the IRE appear to be relevant earlier, with transcript abundance spiking between 9-18 h of iron deprivation. Our new data in **Figure 7** suggests that even this transient stabilisation does have consequences for the parasite in low iron conditions, validating a functional role for the UTR in parasite survival.

How many of the 142 transcripts containing an IRE and regulated by Iron are downregulated?

We have included this in the text. Specifically - ‘Of these, 82 genes were up- or downregulated in the canonical direction, based on 5’ or 3’ UTR positioning, in low iron conditions.’.

The authors only characterized 2 different IREs and only one of them seems functional. It would be better to expand the number of IRE tested to confirm their functionality in this parasite.

We agree and have included qRT-PCR mRNA stability data for a further three transcripts which contain predicted IREs, of which two appear to confer increased stability in low iron. Beyond IREs, our sequencing approach identifies a number of further genes of interest, and potentially new motifs which will be examined in future work.
We agree that this work does not represent a comprehensive assessment of IREs in *Toxoplasma*, however we decided to focus on establishing the kinetics, potential mechanism and functional significance for the first confirmed IRE from the zft 3’ UTR. Future studies will seek to examine the range of IREs and their functionality in more detail, however this initial work seeks to establish a model for studying 3’ UTR regulation in *Toxoplasma*.

Some critical information is missing when exploring the IRE functionality. The distance to the STOP codon could be explored as well as a much-detailed mutagenesis analysis of the IRE. A key experiment is also missing: what happens to the reporter expression when the IRE is placed in the dhfr 3'UTR context? This would help in understanding the role of the motif itself in the observed regulation.

We agree that comprehensive mutagenesis of the IRE and its wider context would be very helpful in understanding the mechanisms behind the transient mRNA stabilisation of our reporter that we see in low iron. However, we believe such an undertaking is beyond the scope of the current work, which seeks to identify this regulation for the first time. Similar studies in mammalian cells have shown that beyond the IREs, multiple other small structures have impacts on responsivity (e.g. Rupani and Connell, 2016 [27307498](https://pubmed.ncbi.nlm.nih.gov/27307498/), Holmes et al, 2019, [31414986](https://pubmed.ncbi.nlm.nih.gov/31414986/)). Given the large size (2143bp) of the UTR and the lack of knowledge about mRNA structures in *Toxoplasma*, we feel that a comprehensive analysis is beyond the scope of this work.

The wider context in which the IRE sits in the transcript can have a large effect on whether it is functional e.g. RNA-folding may mask accessibility of the RNA loop for RNA-binding proteins. Our results here may support this, as the *isu1* mRNA does demonstrate altered stability under low iron, but we did not see this in the reporter we constructed. As such, the interpretation of results would be challenging. We have modified our language in this section to emphasise that positioning is merely one potential aspect in the functionality.

It is rather surprising that the authors choose to use tdTomato as a reporter. The long half-life of the protein and the reduced dynamic range of fluorescence intensity are not fitted to the measurement of transcript stability or translation efficiency. Luciferase is usually used for this purpose. It would be interesting to reiterate some of the experiments with a luciferase reporter to test whether a bias was introduced in the measurements.

We thank the reviewer for their suggestion, we have remade our reporter line with nanoluciferase and show that the reporter responds to iron depletion in a dose dependent manner, confirming our results with tdTomato. The new data is in **figure 3C**

In Figure 5B, the statistical test of the comparison between the tdTomatofit and the tdTomatofit∆IRE is not presented. It seems that the level of expression of the two constructs is not significantly different. If this is the case, this strongly attenuates the importance of the IRE in the response to iron stress.

The reviewer is correct, and we have now highlighted this in the text to make sure this is clear. We agree that the IRE is not the only factor mediating the iron-responsivity of the UTR but we do argue that it contributes to the response that we see, at both the mRNA and protein level. We believe that there are more, currently unknown regions in the UTR (as validated for well understood mammalian systems, e.g. Holmes et al, 2019, [31414986](https://pubmed.ncbi.nlm.nih.gov/31414986/)) however, we demonstrate a contribution for this IRE in our system.

The author failed to demonstrate that Aconitase is binding to the IRE transcripts since actin is also found in the transcripts of the IP fraction. In Figures 6E and 6F, although the rng2 (a cell cycle-dependent transcript) is absent from the IP, the presence of actin shows that the background is high in these experiments. Performing these experiments with more stringent washing steps may be helpful. It would be also more convincing to perform a high throughput sequencing of the transcripts pull-down in the IP to identify the variety of transcripts bound by Aconitase.

We agree and have performed the IP and sequencing as suggested. The new data has been included in **Figure 6**. As the reviewer suggested, we find that the higher stringency does remove signal for zft-binding to aconitase, although interestingly we do see an enrichment of predicted-IRE containing transcripts in the IP. We have now modified our conclusions based on these data.

Reviewer #3: None

**Part III – Minor Issues: Editorial and Data Presentation Modifications**
Please use this section for editorial suggestions as well as relatively minor modifications of existing data that would enhance clarity.

Reviewer #1: Minor issues:
1. “fit” should be defined in abstract and in its first appearance in Introduction. FIT should be worked into the author summary as well since it appears in the title.

We have actually changed the name, from FIT to **Z**n and **F**e **T**ransporter (ZFT). This more closely relates to the predicted function of the protein and we have modified this in the text as below:

*We chose to examine a predicted IRE from the 3’ UTR of an unstudied gene, TGME49_261720, which is predicted to encode a zinc-iron permease, which we named ZFT (zinc and iron transporter).*

2. Many typos and errors in the text (missing punctuation, T. gondii not in italics in all places, extra words, “nt” instead of “not,” etc). Most references are numerals but at least one is a lead author’s name (Augusto 2021) and this paper is not listed in the references section.

We thank the reviewer for their close attention and we have fixed these errors within the text, our apologies.

3. Reword this incomplete sentence: “Selecting a predicted IRE from the 3’ UTR of a predicted, but unstudied iron transporter, TGME49_261720, which we name here FIT.” 4. The authors state in Results: “TGME49_261720 encodes an essential ZIP-domain containing protein (41) and is a putative plasma-membrane localised zinc/iron transporter (35). For these reasons, we renamed the gene FIT.”
Based on this statement, it isn’t clear how the authors derived the name “FIT” – please define exactly what “FIT” stands for; in addition, ZIP should be defined as “zinc/iron permease.”

This sentence has been reworded to improve clarity, see above.

5. This statement reads awkward: “As at the transcript level, there was no significant change in tdTomato fluorescence was observed in the tdTomatodhfr cells at any timepoint.”

We have amended this to read ‘We did not observe any significant change in tdTomato fluorescence was observed in the tdTomato_dhfr_ cells at any timepoint’.

6. A GO analysis of transcripts altered by iron depletion would provide a helpful view of which biological process may be affected by iron deprivation.

We have added this analysis to **Figure S1A and B.**

7. Fig 6 – “localisation was not significantly affected by iron depletion.” Where is this data shown?

**Figure S4B** shows IFA images of the ACN-Ty line cultured in standard culture media and after DFO treatment.

8. Legend contains typos (e.g. CAN-Ty instead of ACN-Ty…damn autocorrect!)

We have fixed these issues, our apologies.

9. It is mentioned that TGME49_261720 is conserved between Toxoplasma genomes; as the RH strain is used for experiments, it seems appropriate to include it in the alignment on Figure S1A.

As the Rh genome is very incomplete, we have added the TGGT1 sequence, from a very closely related type 1 strain which shows that this region is highly conserved.

10. Figure S1B-C: the expected size for each PCR product is not clear in the figure and legends.

This has been added.

11. Fig. 2D: In the manuscript, it is mentioned that there was no increase in the ratio of green:red fluorescence in the tdTomato-dhfr line, but there is p<0.0001 listed on the graph. Did the authors mean to indicate the change as “not significant”?

This was an error, our apologies, and has been corrected in the new version of the manuscript

12. Figure 6G is cited as 6F in the main text and 6H as 6G.

This has been corrected

13. Reference 36 does not appear to have been published in a peer-reviewed journal. Not sure of PLOS policy here.

We no longer cite this paper in the manuscript

14. It would be extremely helpful if authors adhered to a more standardized manuscript format that included more readable font (e.g. arial size 11 or 12), double spacing, and contiguous line numbers for easy referencing.

This has been corrected in the resubmission

Reviewer #2: Minor concerns:
It is not clear which are the 142 transcripts with an IREs identified.

This section has been reworded as follows: Of these, 82 genes were up- or downregulated in the canonical direction, based on 5’ or 3’ UTR positioning, in low iron conditions.’.

The bibliography is sometimes improperly formatted (for ex: Augusto, 2021)

This has been corrected.

Reviewer #3: "supporting a recent observation (36)," sentence fragment?

This has been corrected.

"This suggests that iron deprivation, similar to other nutrient deficiencies (38–40), can promote Toxoplasma differentiation" there is a preprint on this exact topic that would be worth citing if Plos Pathogens allows that.

Luckily, the work mentioned has since been published and we have cited it in our text.

unsure what "loosely targeted effect of iron deprivation" means. I think authors should elaborate.

This has been reworded in the resubmission

fig 2d has P<0.0001 but text says there was no signfiicant difference in the dhfr line. typo?

This was an error and has been corrected

figure 6d,e,f: minor concern about statistical analyses here as I am not seeing anything about how these data were tested statistically or if they were at all.

Information about statistical testing has been added to the main text and figure legends, our apologies.

methods: 3mm should be 3 micrometer? 1 mg should be 1 microgram?

We have corrected these issues, thanks to the reviewer for drawing our attention.
